# Analysis of the Cancer Genome Atlas Data Reveals Novel Putative ncRNAs Targets in Hepatocellular Carcinoma

**DOI:** 10.1155/2018/2864120

**Published:** 2018-06-26

**Authors:** Tiago Falcon, Martiela Freitas, Ana Carolina Mello, Laura Coutinho, Mario R. Alvares-da-Silva, Ursula Matte

**Affiliations:** ^1^Gene Therapy Center, Experimental Research Center, Hospital de Clínicas de Porto Alegre, 90035-903 Porto Alegre, RS, Brazil; ^2^Post-Graduation Program on Genetics and Molecular Biology, UFRGS, 91501-970 Porto Alegre, RS, Brazil; ^3^Graduation Program on Biotechnology/Bioinformatics, UFRGS, 91501-970 Porto Alegre, RS, Brazil; ^4^Gastroenterology and Hepatology Division, Hospital de Clinicas de Porto Alegre, Brazil; ^5^Graduate Program on Gastroenterology and Hepatology, Department of Internal Medicine, Universidade Federal do Rio Grande do Sul, Porto Alegre, RS, Brazil; ^6^Department of Genetics, UFRGS, 91501-970 Porto Alegre, RS, Brazil

## Abstract

Hepatocellular carcinoma (HCC) is the prevalent type of primary liver malignancy. Different noncoding RNAs (ncRNAs) that negatively regulate gene expression, such as the microRNAs and the long ncRNAs (lncRNAs), have been associated with cell invasiveness and cell dissemination, tumor recurrence, and metastasis in HCC. To evaluate which regulatory ncRNAs might be good candidates to disrupt HCC proliferation pathways, we performed both unsupervised and supervised analyses of HCC expression data, comparing samples of solid tumor tissue (TP) and adjacent tissue (NT) of a set of patients, focusing on ncRNAs and searching for common mechanisms that may shed light in future therapeutic options. All analyses were performed using the R software. Differential expression (total RNA and miRNA) and enrichment analyses (Gene Ontology + Pathways) were performed using the package TCGABiolinks. As a result, we improved the set of lncRNAs that could be the target of future studies in HCC, highlighting the potential of* FAM170B-AS1* and* TTN-AS1*.

## 1. Introduction

Epidemiologic data from the International Agency for Research on Cancer of the World Health Organization reveals that liver cancer comprises 5.6% of worldwide cancer incidence and 9.1% of all cancer-associated mortality [1]. Hepatocellular carcinoma (HCC) is the most prevalent type of primary liver malignancy [2]. The high lethality of HCC can be attributed to the lack of diagnostic markers for an early detection and late stages high heterogeneity [3]. HCC has been epidemiologically associated with chronic Hepatitis B Virus (HBV) or Hepatitis C Virus (HCV) [4], as well as alcoholic and nonalcoholic fatty liver disease, which are its major risk factors [2]. Currently, the most effective treatment is either surgical tumor resection or liver transplantation [5].

Multiple studies have shown the potential of different microRNAs (miRNAs) as prognostic and diagnostic biomarkers in many types of cancer, including HCC [6–8]. miRNAs are noncoding RNAs that negatively regulate gene expression by leading mRNAs to target degradation or translational repression after binding to its 3'UTR (for review see [9]). In cancer, their role has been either as tumor suppressors or as enhancers (oncomiRs) [10].

In HCC, different miRNAs have been associated with cell invasiveness by repressing TET gene expression, leading to silencing of several invasion-suppressors via hypermethylation [11], and cell dissemination by regulating differentiation, hence increasing metastatic potential [12]. They are even implicated in improvement of HBV and HCV viral replication and tumor-supporting mechanisms [13, 14]. This multifaceted miRNA capacity of influencing in the HCC environment proves the importance of studies describing expression profiles of miRNAs during tumor occurrence.

Another class of noncoding (nc) RNAs, the long ncRNAs (lncRNAs), are > 200 nucleotides' RNA molecules with multiple regulatory roles that can not be inferred by their sequence. These roles comprise, among others, chromatin organization affecting the gene expression [15].* HOTAIR*, an antisense lncRNA, has been associated with HCC recurrence and metastasis [16].* HULC* and* FTX* (HCC) are also upregulated in tumoral samples [17].

Here, differently from previous works that focused on viral infection (HBV or HCV) comparing primary solid tumor tissue (TP) and adjacent tissue (NT) [6, 8, 18, 19], or focused on the mutation found [20], we* in silico* compared TP and NT of a set of patients in sense to discover the pathways that differentiate both groups of samples and the regulatory ncRNAs and their putative targets. As a result, we improve the set of lncRNAs that could be the target of future studies.

## 2. Material and Methods

All analyses were performed using the R software (v. 3.4.0) [21]. The differential expression (mRNA and miRNA) analysis was performed using the package TCGABiolinks (v. 2.7.1) [22]. First, we downloaded HCC harmonized data (hg38) from The Cancer Genome Atlas (TCGA) using the function* GDCdownload* with the option* legacy = FALSE*. We analyzed a total of 41 participants that have expression data of both primary solid tumor and adjacent tissue samples. It is worth noticing that in the database adjacent tissue is referred to as normal; however this is hardly the case as all patients were cirrhotic. Thus we use the term adjacent, as this is not a sample from a normal liver. To select these individuals, we used only the participant ID of the TCGA barcode as query barcode (e.g., participant ID in bold:** TCGA-BC-A10Q**-01A-11R-A131-07). The sampling comprises a group of men and women, white, black, or Asian, showing or not the presence of risk factors such as fat liver disease. Not all samples had a positive diagnostic for HBV or HCV. All data is available at TCGA web portal.

For total RNA differential expression, we followed the standard pipeline. The samples were highly correlated after an outlier check (*TCGAanalyze_Preprocessing* function). Except one sample (0.85 < r < 0.9) all other samples showed an r > 0.9. Then, we followed a normalization step using both GC content and gene length (*TCGAanalyze_Normalization*) and gene filtering by quantile (*TCGAanalyze_Filtering*) as recommended in [23]. Differentially expressed genes (DEGs) were accessed by the function* TCGAanalyze_DEA* considering a log2 fold change (logFC) of > 1 or < -1. and false discovery rate (FDR) of 0.01. Enrichment analyses of DEGs and top 10 categories' plot were performed by the functions* TCGAanalyze_EAcomplete* and* TCGAvisualize_EAbarplot*, respectively.

Heat maps were plotted using the function* heatmap.2* from package gplots (v. 3.0.1) [24] considering the gene expression information of the top genes based on significant FDR or all differentially expressed transcripts of the categories miRNA, precursor microRNA (pre-miRNA), and lncRNA. Hierarchical cluster analyses were performed using the package pvclust (v. 2.0-0) [25] with 1000 bootstrap replications. Clusters with approximately unbiased grouping support p values (%) (au – red values) of 95 were considered as statistically significant groups.

For the differentially expressed transcripts, we performed a Spearman correlation to detect which regulatory RNAs are negatively correlated with other RNAs. We accepted those with r < -0.8 and p value < 0.05 as statistically significantly correlated. These correlated transcripts were used as interactions to input the network on Cytoscape (v. 3.5.1) [26], where the edges represent the statistically significant r values. The miRNAs and their putative targets were used to predict their interaction using the online software TargetScan (release 7.1) [27]. Interactions not found in TargetScan were also tested in miRDB [28] and TarBase (v. 8) [29]. The interactions found by either TargetScan or TarBase were confirmed by two other tools: miRWalk v. 3.0 (http://mirwalk.umm.uni-heidelberg.de/) [30] considering a binding probability cut-off of 0.8, and mirDIP v. 4.1 (http://ophid.utoronto.ca/mirDIP/index.jsp) [31, 32] considering a “medium” cut-off of scores. Gene Ontology Biological Processes of the proteins associated with the network were evaluated using the Cytoscape plugin BiNGO [33]. For the interest in lncRNAs, we performed a supervised prediction model using the Area Under the Curve of the Receiver Operating Characteristic (AUC-ROC) using the package pROC v. 1.11.0 [34].

## 3. Results and Discussion

In this study we performed a supervised analysis of HCC expression data focusing on ncRNAs searching for common mechanisms that may shed light in future therapeutic options. The majority of statistically significant differentially expressed ncRNAs are higher expressed on tumor samples, suggesting that these RNAs are necessary to tumor progression/maintenance. Additionally, tumor samples showed a more diverse expression profile in comparison to those from adjacent tissues. Such pattern has been reported also for gastric [35] and colorectal cancers [36].

**Figure 1:**
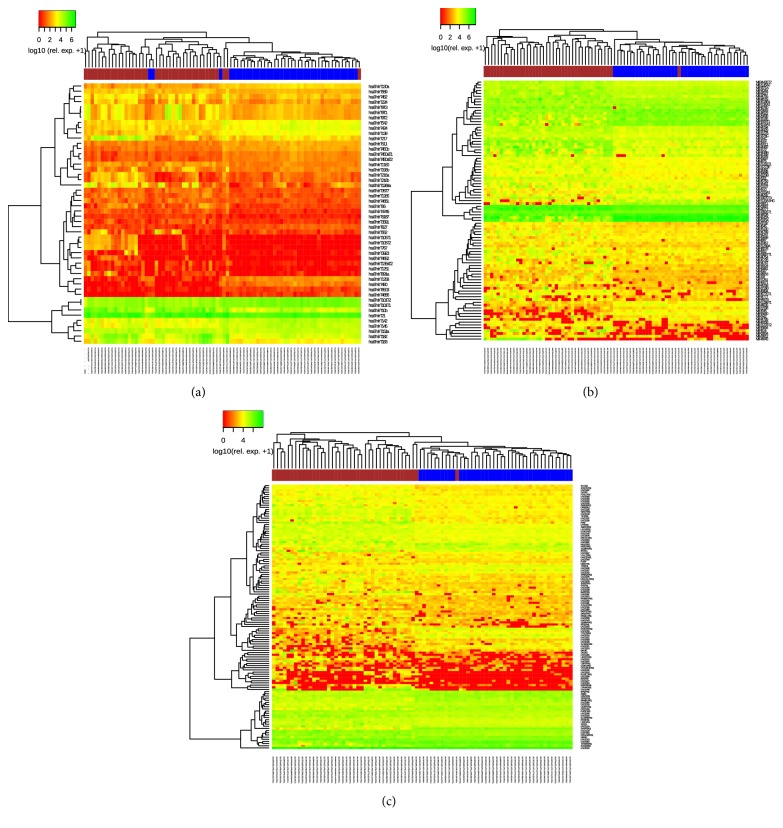
Heat maps of differentially expressed noncoding regulatory RNAs. (a) miRNA. (b) Pre-miRNA. (c) LncRNA. Tumor samples in brown and adjacent samples in blue. Hierarchical clusterization based on transcript log10 scale expression.

We found a total of 1739 DEGs in total RNA-seq among tumor and normal samples. From these, 1276 were upregulated in tumor (Figure 1(a), Figure S1A, and Table S1). miRNA differential expression (DE) revealed 234 DE miRNAs, of which 169 were upregulated in tumor (Figure 1(b), Figure S1B, and Table S1). Other noncoding regulatory RNAs resulted in 92 pre-miRNAs (73 upregulated in tumor) and 122 lncRNAs (90 upregulated in tumor) (Figure 1(c), Table S2). Considering the fold change of DEGs and DE miRNAs, the top ten up- and downregulated genes in tumoral samples are shown in Table 1.

**Figure 2:**
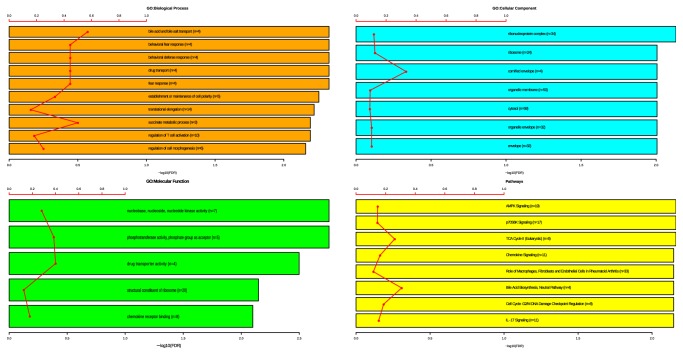
Enrichment analysis of differentially expressed transcripts from total RNA data. GO: Gene Ontology. The red lines represent the ratio of genes found for the pathway over the total number of genes for that specific pathway. Inside each bar, n: number of genes. Bar sizes are in agreement to the -log10 of the FDR of the enriched ontology/pathway.

**Figure 3:**
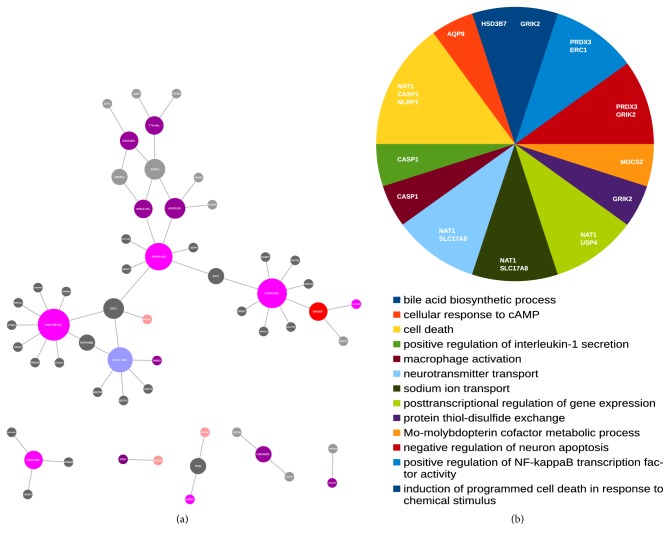
Representative network of the negative Spearman correlation of ncRNAs and putative affected target interactions and Gene Ontology Biological Processes. (a) Representative network of Spearman's negative correlation. Each edge represents an r < -0.8 and p value <0.05. Light blue: downregulated miRNAs in TP samples. Red: upregulated pre-miRNAs in TP samples. Light red: downregulated pre-miRNAs in TP samples. Purple: upregulated lncRNAs in TP samples. Light purple: downregulated lncRNAs in TP samples. Dark gray: upregulated heterogeneous RNAs in TP samples. Light gray: downregulated heterogeneous RNAs in TP samples. Nodes' size represents the degree of connectivity. (b) Gene Ontology Biological Processes with statistically significant (FDR < 0.05) representation. The name of the proteins enriching each process is inside the respective piece of the pie plot.

The enrichment analysis (Gene Ontology + Pathways) revealed that the most represented pathways in differentially expressed transcripts from total RNA-seq are involved in bile metabolism, fear behavioral response, and immune-related categories (Figure 2). To infer putative expression relationship, we plotted a network based on Spearman's correlation, considering only the negative interactions. These interactions involved a total of 18 highly correlated regulatory ncRNAs of all types (miRNA, pre-miRNA, and lncRNA) with their putative targets (Figure 3(a)). In the case of miRNAs and pre-miRNAs, the miRNA-target interactions were predicted as explained in the Material and Methods. These highly negative targets are most involved in programmed cell death, immune response, and Molybdenum cofactor biosynthesis processes (Figure 3(b)). For the lncRNAs in the network, we calculated the AUC-ROC values and found four lncRNAs with potential to correctly discriminate TP and NT samples:* CCND2-AS1* (AUC = 0.792, 95% confidence interval: 0.6834-0.8903),* FAM170B-AS1* (AUC = 0.917, 95% confidence interval: 0.8387-0.9758),* TTN-AS1* (AUC = 0.901, 95% confidence interval: 0.84-0.9539), and* SYNPR-AS1* (AUC = 0.939, 95% confidence interval: 0.8798-0..9823).

The DEGs' enrichment analysis suggested that bile metabolism and fear behavioral response immune-related categories are the most represented pathways. Immune-related categories are usually disrupted in cancer. For example,* CD274*, upregulated in our TP samples, confers immune resistance to tumor cells by the inactivating cytotoxic T-cell [37].* DACT1*, which encodes for an antagonist of beta catenin 1, and* DVL2*, a dishevelled protein family member, are respectively, down- and upregulated in tumor tissues, suggesting that the Wnt signaling is active [38]. Additionally,* CDK14* and* GSK3B* are upregulated, reinforcing the Wnt signaling activation, which is related to cell polarity category [39]. This signaling pathway has been associated with malignant transformation [40].

GO group classified as fear behavioral response includes a series of genes neurotransmitters (such as glutamate, dopamine, and serotonin receptors), which comes as no surprise since many studies have shown the impact of serotonin, GABA, and sympathetic neurotransmitters in hepatocyte proliferation [41–43]. It also includes MECP2 and transcription factors associated with chromatin remodeling. Finally, bile acids are also known to act as potential carcinogens and deregulation of bile acids homeostasis has been linked to HCC formation [44].

Another transcription factor, FXR2, is supposed to act as heterodimer (or larger complexes) with TP53 or FXR1, suppressing tumor development. However,* TP53* and* FXR1* expressions were not detected after normalization process. Still, FXR2 can interact as homodimer or as a larger complex [45]. The absence of* FXR1* expression could be a consequence of* GSK3B* upregulation, once FXR1 phosphorylation by GSK3B leads to* FXR1* downregulation [46].

From the negative correlation network (Figure 3), we can highlight the immune-related categories, as occurred in the DEGs enrichment analysis. The expression of* NAT1* has been recently reported to show high expression in breast cancer and be associated with steroid biosynthetic pathway genes [47]. Here,* NAT1* is also upregulated in TP samples. This gene's expression is also negatively correlated with two lncRNAs, both antisense RNAs:* SYNPR-AS1* and* CCND2-AS1*.* CCND2-AS1* is known to promote glioma cell proliferation by activating Wnt/*β*-catenin signaling [48], but it is downregulated in HCC.* CASP1*, usually downregulated in cancer cells once it promotes apoptosis [49], showed a high expression pattern in TP samples and is negatively correlated with* CCND2-AS1*.* NLRP1* expression is associated with tumor inflammasomes and suppression of apoptosis in metastatic melanoma [50]. However,* NLRP1* is downregulated in our tumor data and negatively correlated with the pre-miRNA MIR3667. This miRNA is known to disrupt the oncogenetic activity of* PCAT-1*/MYC in prostate cancer [51] and thus its low expression in TP samples is expected.* GRIK2*, correlated with tumor progress [52],* ERC1*, which is upregulated in TP samples and its expression is associated with tumor progression once it is necessary to focal adhesion turnover [53], and* PRDX3*, whose overexpression is highly connected to prostate cancer [54] by protecting tumoral cells from oxidative stress [55], are all upregulated in TP samples of HCC and negatively correlated with the expression of the antisense RNA* FAM170B-AS1*.* ERC1* is also negatively correlated with* SYNPR-AS1*, hsa-mir-139, which is known to play antitumoral roles in HCC [56], and the pre-miRNA MIR320A plays antitumoral roles in breast cancer [57]. SLC17A8 is upregulated in prostate cancer [58] and in HCC and negatively correlated with* SYNPR-AS1*.* HSD3B7* is associated with bile acid and did not change its expression in* CTNNB1* mutated HCC samples [59]. However, here* HSD3B7* is upregulated in TP samples and negatively correlated with* LINC01493*.

NLRP1/CASP1 form a complex that induces pyroptosis [60], a cell death dependent on CASP1 and associated with many pathological conditions, including cancer [61]. Bearing in mind that we can not interpret gene expression as active protein production or enzymatic activity, still it seems like pyroptosis pathway is disrupted in HCC in comparison to other cancer types and that* CCND2-AS1* might play a role by regulating* CASP1* expression in this process.

*AQP9* overexpression decreased the PIK3CB levels in normal tissues, reducing the cell proliferative potential by increasing FOXO1 levels and reducing PCNA expression [62]. In HCC, AQP9 levels are low [63] inducing PIK3CB activity and cell proliferation [62]. In agreement with these authors,* AQP9* is downregulated in our TP samples profile, while* PIK3CB* is upregulated.* PCNA* is also upregulated but did not pass the logFC cut-off.* AQP9 *is negatively correlated with* TTN-AS1*, which was recently described as an oncogene highly expressed in esophageal squamous cell carcinoma progression and metastasis [64].

Hierarchical cluster analysis of the differentially expressed total transcripts, miRNAs, pre-miRNAs, and lncRNAs, shows that statistically significant groups are created in all cases, discriminating most adjacent from tumoral samples. This kind of distinction was not found when trying to differentiate samples also by viral types (HBV or HCV) (data not shown). It is worth noticing that DEG, but especially ncRNA analysis, was able to perfectly discriminate between TP and NT, although it was not able to separate HBV and HCV-infected samples. This suggests that the mechanisms depicted here are common to HCC regardless of its causative injury. Even though risk factors for HCC are well-known, it remains as an important cause of death worldwide. Although tumor surveillance in cirrhotics is highly recommended by international guidelines [65], late diagnosis is quite common. Moreover, advanced liver disease and parenchymal dysfunction further prevent curative therapies [66].

## 4. Conclusions

Our data suggests that neither HBV nor HCV infection changes overall gene expression (including those genes encoding for ncRNAs) in TP samples. Pyroptosis pathway is misregulated in HCC if compared to other cancer types and the lncRNA* CCND2-AS1* might be involved in this misregulation, revealing a singular characteristic of HCC. Additionally,* FAM170B-AS1* and* TTN-AS1* emerge as new candidates to tests to disrupt HCC homeostasis by turning cancer cells susceptible to oxidative stress or affecting cancer cell proliferation, respectively. Also, these lncRNAs show remarkable expression signatures, differentiating TP from NT samples with high AUC-ROC values.

## Figures and Tables

**Table 1:** Top ten differentially expressed genes (DEGs) from total RNA-seq and microRNA RNA-seq.

Total RNA-seq DEGs			
Upregulated in tumor		Downregulated in tumor	
Transcript	Fold change	Transcript	Fold change
SIRT1	15.32119	PAPPA2	-5.66486
RPL28	11.29295	TNR	-4.59590
LINC01613^†^	10.83190	SNHG21	-4.51615
CACNB3	10.71749	CALHM6	-4.44294
CREM	10.54143	LCMT1-AS2^†^	-4.39991
GOLGA8B	10.32588	BHMT2	-4.34723
SLC46A2	10.16996	PRDM1	-4.33354
LINC00449^†^	10.10149	CPLX4	-4.29631
NUP85	9.78697	PTP4A2	-4.06166
CFAP44-AS1^†^	9.67638	GEMIN4	-3.99574

DE miRNAs			
Upregulated in tumor		Downregulated in tumor	
Transcript	Fold change	Transcript	Fold change

hsa-mir-767	8.99037	hsa-mir-490	-3.58293
hsa-mir-105-2	8.90799	hsa-mir-4686	-3.40137
hsa-mir-891a	8.86653	hsa-mir-1258	-2.95466
hsa-mir-105-1	8.19189	hsa-mir-139	-2.00361
hsa-mir-3923	7.94124	hsa-mir-424	-1.96325
hsa-mir-520f	7.91529	hsa-mir-4683	-1.94045
hsa-mir-520c	7.63784	hsa-mir-934	-1.92190
hsa-mir-518e	7.37922	hsa-mir-130a	-1.86453
hsa-mir-520b	7.17289	hsa-mir-873	-1.84610
hsa-mir-520a	7.00665	hsa-mir-6503	-1.76457

^†^putative regulatory lncRNA.

## Data Availability

All data used in this work is publicly available at The Cancer Genome Atlas (TCGA) database <https://cancergenome.nih.gov/>.

## Supplementary Materials

Supplementary Materials Figure S1: volcano plot representing differentially expressed transcripts (primary solid tumor x normal tissues). (A.) Total RNA. (B.) MicroRNA. Blue dots: upregulated transcripts in tumor samples. Red dots: downregulated transcripts in tumor samples. Black dots: statistically nonsignificant expressed transcripts. logFC: log2 fold change. Vertical gray lines: cut-off of logFC < -1 and logFC > 1. Horizontal purple line: FDR cut-off of 0.01. Table S1: differentially expressed data from total RNA. logFC: log2 fold change. Table S2: differentially expressed miRNAs. logFC: log2 fold change.
